# Development and External Validation of a Machine Learning–Based Risk Score for Stent Outcomes in Post–Bariatric Leak Management: The “Alexandria-Bari-Stent” Tool

**DOI:** 10.1007/s11695-025-08321-6

**Published:** 2025-11-29

**Authors:** Mohamed Hany, Ehab Elmongui, Walid El Ansari

**Affiliations:** 1 Alexandria University, Alexandria, Egypt; 2 Madina Women’s Hospital, Alexandria, Egypt; 3 Independent Biostatistical Consultant, Alexandria, Egypt; 4 Ajman University, Ajman, United Arab Emirates

**Keywords:** Bariatric surgery complications, Endoscopic stent, Gastric bypass, Sleeve gastrectomy, Leak, Perforation, Artificial intelligence

## Abstract

**Background:**

There are no prediction models of stent outcomes for leaks after metabolic and bariatric surgery (MBS). The current study developed an artificial intelligence–based model to predict post-MBS stent failure.

**Methods:**

Prospectively maintained database of patients with post-MBS leaks was used for model development (Center I, *N* = 250); external validation employed patients from another hospital (Center II, *N* = 150). Outcome definition was failure of the first (primary/initial) stent implantation to resolve the leak, i.e., lack of primary closure. Ranking of variables was performed, 11 machine learning algorithms were tested, the best model was selected, and a stent failure point-based risk scoring system was derived, with further external validation, calibration, and decision curve analysis.

**Results:**

The development cohort (training sample, Center I) had 27.6% failed stents/72.4% successes; the external validation cohort (Center II) had 30% failures/70% successes. The Lasso logistic regression model exhibited the best performance. Eight variables contributed to the model’s predictive performance (obstructive sleep apnea, hypertension, diabetes, hepatomegaly, hyperlipidemia, body mass index, Niti-S18 stent, gastrojejunal anastomosis leak), and nine others had varying contributions (revisional surgery, Niti-S23 stent, time to stent implantation, leak size > 1 cm, age, Roux-en-Y gastric bypass surgery, esophagogastric junction leak, Hanaro 21 stent, male sex). The clinical point-based stent failure risk system showed that scores ≤ 7 had very low failure risk (<1%), scores 8–47 = low risk (1–5%), 48–77 = moderate risk (5.1–15%), 78–117 = high risk (15.1–50%), and scores ≥198 were associated with extremely high failure risk (>96%). The model’s external validation demonstrated excellent discriminatory power, distinguishing between patients with/without the outcome with 0.85 area under the ROC curve (95% CI: 0.76–0.93), 80% sensitivity (95% CI: 65.4-90.4%), 82.9% specificity (95% CI: 74.3-89.5%), and 66.7% positive predictive value (95% CI: 52.4–79.0%). The negative predictive value was 90.6% (95% CI: 82.9–95.6%) indicating that the model was particularly effective at identifying patients unlikely to fail. Area under the precision-recall curve was 0.81 (95% CI: 0.70–0.89) indicating strong performance in identifying true positives while minimizing false positives. Calibration was acceptable (Brier score = 0.15). Decision curve analysis demonstrated higher net benefit when used in clinical decision-making across a broad range of threshold probabilities (0.10–0.80) compared to treating all patients or treating none.

**Conclusions:**

A machine learning model (Alexandria-Bari-Stent) can predict post-MBS stent failure. External validation displayed high accuracy, good sensitivity/specificity, and excellent negative predictive value indicating good discriminative ability. Clinically, the model is more reliable for ruling out stent failure than confirming it, making it especially useful in reassuring low-risk post-MBS leakage patients. Patient’s general status, metabolic health, and systemic factors appeared to play a more critical role than previously recognized, complementary to, not in conflict with, established technical and local factors that influence successful stent outcomes for leak management. This prompts the need for a more holistic view of leak patients who are candidates for stenting. Prospective multicenter trials are needed to confirm the performance of the Alexandria‑Bari‑Stent model and the role of metabolic stabilization and medically optimizing the patient for better outcomes.

**Supplementary Information:**

The online version contains supplementary material available at 10.1007/s11695-025-08321-6.

## Introduction

Metabolic and bariatric surgery (MBS) is currently the most effective long-term treatment of severe obesity with or without associated medical conditions. Despite the low rates of complications after MBS, gastrointestinal leaks are concerning adverse events, with levels of <1.3% and <0.15% after primary Roux-en-Y gastric bypass (RYGB) and sleeve gastrectomy (SG), respectively, and a 2.4% risk for all new MBS [1, 2]. Leakage remains an important cause of post-MBS morbidity and mortality [3–5].

Risk factors associated with leak include higher BMI, staple height and use of buttressing material did not affect leak rate, size ≥40-Fr bougie was associated with less leak, and longer time to referral and lower serum prealbumin level were independently associated with poorer evolution of post-SG gastric leak under conservative management [6].

Management of post-MBS leaks involves surgical, endoscopic, and/or radiological options [7]. Surgery is associated with significant morbidity/mortality, endoscopy offers significant benefits for selected patients [8, 9], and stent placement is less invasive than surgery. A challenge in managing post-MBS leaks is the scarcity of well-designed studies to guide evidence-based treatment [10].

The literature reveals knowledge gaps. Stents seal the defect and divert luminal content, allowing mucosal wall healing, early oral intake, and reduced stricture formation [11]. However, post-MBS stent research recruited heterogeneous patients, examined stent efficacy for a variety of indications, lacked standardization, and revealed practice discrepancies, and several classification systems for post-MBS leaks exist, with no universal adoption or comprehensive validation [12]. Hence, endoscopic techniques used to manage upper gastrointestinal anastomotic leaks lack consensus on the most appropriate therapeutic approach [13] and no solid evidence to guide optimal stent treatment for the best outcomes [10, 14]. With such uncertainty, a predictive tool is needed to identify the patients with post-MBS leaks who are likely to fail stent therapy. This is important, as leaks are life-threatening [15, 16].

Anticipating the risk of stent failure is critical to individualized treatment approaches that involve selection of optimal patients, MBS type, BMI category, associated medical conditions, leak site/size, and stent type/length. A one-size-fits-all approach does not exist, and successful management requires a tailored approach premised on clinical parameters, surgical features, local expertise, and availability of devices [17].

Hence, a clinically feasible stent failure risk prediction model is critical to accurately forecast stent failure risk for post-MBS leaks, devise preventive strategies, and reduce mortality. To the best of our knowledge, no previous research undertook such a task.

Therefore, the aim of this study was to develop and externally validate a machine learning (ML)–based risk model (Alexandria-Bari-Stent) to predict stent failure in post-MBS leaks. The specific objectives were to

1. Develop a clinical predictive model for post-MBS stent outcomes employing patients from one MBS center (development sample).
2. Identify the demographic, surgical, clinical, leak, and stent-related variables associated with stent outcomes (predictors).
3. Compare the performance of ML algorithms and select the most appropriate model.
4. Evaluate the final model’s performance on an external validation dataset.
5. Assess the model’s permutation-based feature importance.
6. Convert the model’s coefficients into a point-based scoring system for predicting stent outcomes.
7. Externally validate the risk score model on another 150 patients (external validation sample).
8. Calibrate the performance of the point-based risk score model in the external validation dataset.
9. Appraise the model’s clinical utility using decision curve analysis (DCA).

We also sought to generate a flowchart of the management outcomes of the 400 post-MBS leakages.

## Materials and Methods

### Study Design and Ethics

We retrospectively reviewed the data of all consecutive patients who underwent endoscopic stent placement for staple line and anastomotic leaks after primary (predominantly SG or RYGB) or revisional MBS (lap band or vertical banded gastroplasty converted to SG or RYGB) during the study period. Patients’ medical history, perioperative information, stent placement details, outcomes, and subsequent interventions were analyzed. The Research Ethics Committee at the two participating institutions approved the study (approval number: IORG0008812; E/C.S/N.R2/2023), which was reported using the TRIPOD guidelines (and TRIPOD-ML extension) [18].

### Settings: Participating Centers

The participating MBS centers were two separate university-based centers in different hospitals in Alexandria, Egypt. The development (training) set employed to establish the model comprised all consecutive patients with post-MBS stent placement at Center I. The external validation included all consecutive patients with post-MBS stent placement at Center II during the same period. Both cohorts had the same inclusion criteria, namely, patients who underwent endoscopic stent for post-MBS leak between January 2015 and September 2024.

### Patient Populations

*Development (training) set*: 250 consecutive patients with post-MBS leaks who had stents inserted during the study period, referred to Center I from private hospitals (*n* = 200) or had MBS at the same center (*n* = 50) and managed primarily by fully covered self-expandable metallic stents (Niti-S MEGA Esophageal Stent, Taewoong Medical, Gyeonggi-do, South Korea). Patients managed by other endoscopic modalities were excluded, e.g., internal drainage (*n* = 43), Ovesco clip (n = 26), or those who experienced failure to deploy the stent due to inability to introduce the guidewire (*n* = 4).

*External validation set*: 150 consecutive patients with post-MBS leaks at Center II during the same time period, referred from hospitals from neighboring governorates (*n* = 106) or who were from the same center and developed leaks (*n* = 44) and were managed by the same types of stents as in Center I. Patients managed by endoscopic modalities other than stent were excluded (*n* = 56) or those who experienced failure of stent insertion (*n* = 7).

### Endoscopic Stenting

Box 1 depicts the steps undertaken. Consultant endoscopists performed the stenting, where catheters were embedded over a guidewire and stents were deployed under fluoroscopy guidance, with adequate positioning of the stent confirmed by endoscopy. Endoscopic stents were selected based on leak characteristics and anatomical considerations. While concurrent strictures were managed as clinically indicated, systematic documentation of balloon dilation procedures was not performed in this retrospective study. The self-expandable metal stents used (Niti-S and Hanaro) provide both leak sealing and stricture dilation functions simultaneously.

**Box 1** Endoscopic stenting: technique and insertion

**Table:** 

Step	Description
Guidewire placement	Endoscopic guidewire introduced via endoscope’s working channel, advanced distally, monitored under C-arm guided fluoroscopy
Stent insertion	Using guidewire, collapsed self-expanding metal stent (Niti-S) advanced to desired position within gastric tube, ensuring stent covered entire leak site and proximal and distal margins
Stent expansion	Stent gradually released from delivery sheath, allowing it to expand and adapt, ensuring proper placement
Verification and positioning adjustment	Endoscopic examination verified stent’s correct placement and coverage of leak site. Fluoroscopy confirmed positioning. Contrast study conducted by injecting water-soluble contrast through the scope, ensuring that leak was sealed
Final evaluation and monitoring	Concluding evaluation verified stent’s correct positioning and operation. Post-procedure, patients were observed for possible complications; follow-up care was scheduled. Stents were monitored periodically by X-rays and endoscopic sessions, and computed tomography

### Outcome Definitions

As elsewhere [1, 17, 19], success was defined as radiographic and endoscopic confirmation of leak closure after stent therapy (e.g., evidence of no contrast extravasation once the stent is removed, gastro-intestinal continuity). Failure was persistence of leak despite stent placement, requiring alternative intervention such as repeat endoscopy or surgical treatment. In the current study, success/failure rates were calculated based on the success/failure of the first (primary/initial) stent placement to resolve the leak.

### Data Collection

Data extracted from patients’ records included data on demography, surgery, leak, stent, associated medical conditions, outcomes, and health system–related information (Box 2).

**Box 2** Data extracted from patients’ records

**Table:** 

Data	Description
Demographic	Age, sex, body mass index
Associated medical condition/s	Obstructive sleep apnea, diabetes mellitus, hepatomegaly, hyperlipidemia, hypertension, osteoarthritis, polycystic ovary syndrome [20]
Surgery related	History of previous operations, type of MBS procedure, whether MBS was primary or revisional
Leak related	Site of leakage, size of leak (radiology and endoscopy)
Stent related	Stent length, time to stent from relevant surgery, duration of stent placement, stent-related complications
Outcomes	Stent success, failure
Health system related	Length of overall hospitalization, number of readmissions, mortality

Leak was diagnosed from clinical signs that included persistent fever, tachycardia, dyspnea, persistent abdominal/left shoulder pain, radiographic evidence (extravasation of oral contrast or intra-abdominal abscess) with or without necessity for radiology-guided drainage of intra-abdominal collection, and endoscopic evidence of staple line disruption [21]. The lack of a universally accepted validated classification system [22–25] during our study period (2015–2024) represented a limitation.

### Post-stenting Nutritional Management Protocol

Nutritional management varied across the two centers and was customized based on individual patient factors. Typically, patients were initially kept NPO for 24–48 h after stenting with IV fluid support. The choice of subsequent nutritional support depended on clinical factors such as leak severity, patient stability, the expected duration of bowel rest, and patient tolerance. Management strategies included advancing the oral diet gradually for stable patients with contained leaks and a stent in place, as this enables early enteral nutrition once patients can tolerate it; placing a gastrojejunal feeding tube for patients requiring extended enteral nutrition who were not adequately ingesting nutritional supplements; and using total parenteral nutrition (TPN) for patients who could not tolerate enteral support for a prolonged period or had a non-functioning GI tract.

### Statistical Analysis

Statistical analyses were performed using R software version 4.4.2. Descriptive statistics, including means, standard deviations, medians, ranges, frequencies, and percentages, summarized the demographic and clinical characteristics of the study cohorts. Chi-square tests for categorical variables and independent *t*-tests or Mann–Whitney *U* tests for continuous variables assessed any differences between the development and validation samples. Details pertaining to variable selection for model creation, data preprocessing for machine learning, and the machine learning process are depicted in Supplementary Box 1. The Lasso logistic regression was selected as the final model.

## Results

**Fig. 1:**
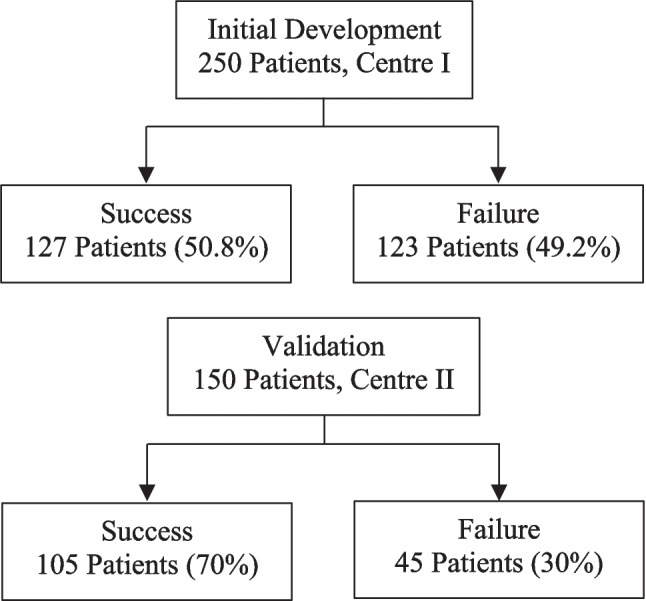
Flowchart depicting progression and outcomes of clinical predictive model development and validation for stent failure for leak among post-MBS patients. Model was initially developed employing 250 patients, resulting in 127 successful cases on first attempt (50.8%) and 123 failures (49.2%). It was then validated with a cohort of 150 patients from a different center, exhibiting 105 successes (70%) and 45 failures (30%)

The numbers of patients used in the development and validation of the clinical predictive model for stent failure are depicted in Fig. 1. Employing a sample of 250 patients from Center I, 123 cases (49.2%) resulted in failed stent, and 127 cases (50.8%) were successful. The model was then validated using a cohort of 150 patients from Center II, which had 45 cases (30%) of failure and 105 successful cases (70%).

### Baseline Characteristics of Model Development (Training) Cohort

Patients included in the model development (training) sample were aged about 44.3 years, with 68.4% females and a mean body mass index (BMI) of 47.2 kg/m^2^ (Table 1). Most underwent primary MBS, specifically sleeve gastrectomy. Associated medical conditions comprised OSA (34%), diabetes (33.6%), and hepatomegaly (32%). All patients presented with fever and raised C-reactive protein levels. Most leaks occurred at the esophagogastric junction (EGJ, 86.4%), and the predominant stents used were Hanaro 21 cm and Niti-S 23 cm. Average time to stent placement was 20.3 days, with a mean duration of stenting of 21.8 days. The stent failure rate was 49.2%; mortality was 1.2%.

**Table 1:** Characteristics of patients with post-MBS leakage used in model development of stent outcomes (*N* = 250)

Variable	Value
Demography	
Age (years)	
M ± SD	44.3 ± 8.2
Range	27–57
Sex	
Male	79 (31.6)
Female	171 (68.4)
Surgery	
Type	
Primary	220 (88)
Sleeve gastrectomy	201 (80.4)
Roux-en-Y gastric bypass	49 (19.6)
Revisional	30 (12)
Vertical banded gastroplasty	15 (6)
Sleeve gastrectomy	9 (3.6)
Lap band	5 (2)
Plication	1 (0.4)
Clinical	
Body mass index (kg/m^2^)	
M ± SD	47.2 ± 3.6
Range	41–54
Associated medical conditions	
Obstructive sleep apnea	85 (34)
Diabetes mellitus	84 (33.6)
Hepatomegaly	80 (32)
Hyperlipidemia	73 (29.2)
Hypertension	63 (25.2)
Osteoarthritis	6 (2.4)
Polycystic ovary syndrome	2 (0.8)
Presenting symptom/s	
Fever	250 (100)
Raised C-reactive protein	250 (100)
Pain	128 (51.2)
Pleural effusion	122 (48.8)
Leak	
Site	
Staple line leaks	
Distal staple line (SG)	3 (1.2)
Esophagogastric junction	216 (86.4)
Esophagogastric junction + distal staple line (SG)	1 (0.4)
Anastomotic leaks	
Gastrojejunal anastomosis	30 (12)
Size	
<1 cm	69 (27.6)
>1 cm	181 (72.4)
Stent	
Time to stent placement (days), M ± SD	20.3 ± 6.2
Duration of stent (days), M ± SD	21.8 ± 6.9
Type	
Niti-S	
23 cm	100 (40)
18 cm	24 (9.6)
Hanaro	
18 cm	25 (10)
21 cm	101 (40.4)
Outcome	
Success	127 (50.8)
Failure	123 (49.2)
Surgery	5 (2)
Hospitalization, overall (days), M ± SD	3.4 ± 2.4
Number of readmissions, median (range)	1 (1–4)
Mortality	3 (1.2)

Cell values represent frequency and percentages *n* (%) unless otherwise stated, *M* ± *SD* mean ± standard deviation, *SG* sleeve gastrectomy

### Univariable Analysis of Factors Associated With Stent Outcomes

Table 2 shows that demographically, males (OR = 8.10), older age (OR = 1.05 per year), and higher mean BMI (OR = 1.08 per BMI unit) were significantly associated with failure. No significant associations were noted for index surgery type or primary vs. revisional surgery. Prior SG and plication were reported only among failure cases. Other previous surgeries, e.g., vertical banded gastroplasty and lap band, were infrequent and not associated with failure.

**Table 2:** Factors associated with stent failure among model development sample

Predictor	Stent		Simple logistic regression	
	Success (*n* = 127)	Failure (*n* = 123)	Odds ratio (95% CI)	*p*
Demography				
Sex				<*0.001*
Female	112 (88.2)	59 (48)	Reference	
Male	15 (11.8)	64 (52)	8.10 (4.35, 15.90)	<*0.001*
Age, years M ± SD	42.6 ± 8.9	46 ± 7.1	1.05 (1.02, 1.09)	*0.001*
BMI, kg/m^2^ M ± SD	46.7 ± 3.5	47.7 ± 3.6	1.08 (1.01, 1.16)	*0.029*
Surgery				
Index surgery				
Sleeve gastrectomy	100 (78.7)	101 (82.1)	Reference	0.502
Roux-en-Y gastric bypass	27 (21.3)	22 (17.9)	0.81 (0.43, 1.51)	
Type of surgery				
Primary	114 (89.8)	106 (86.2)	Reference	0.385
Revisional	13 (10.2)	17 (13.8)	1.41 (0.65, 3.09)	
Previous surgery				
Sleeve gastrectomy	0 (0)	9 (7.3)		
Vertical banded gastroplasty	10 (7.9)	5 (4.1)	0.50 (0.15, 1.44)	0.213
Lap band	2 (1.6)	2 (1.6)	1.03 (0.12, 8.72)	0.974
Plication	0 (0)	1 (0.8)		
Associated medical conditions				
Obstructive sleep apnea	20 (15.7)	65 (52.8)	6.00 (3.36, 11.08)	<*0.001*
Hepatomegaly	26 (20.5)	54 (43.9)	3.04 (1.75, 5.38)	<*0.001*
Diabetes	28 (22)	56 (45.5)	2.96 (1.72, 5.17)	<*0.001*
Hypertension	24 (18.9)	39 (31.7)	1.99 (1.12, 3.61)	*0.021*
Hyperlipidemia	29 (22.8)	44 (35.8)	1.88 (1.09, 3.30)	*0.025*
Polycystic ovary syndrome	0 (0)	2 (1.6)		
Osteoarthritis	0 (0)	6 (4.9)		
Leak				
Size				<*0.001*
< 1 cm	108 (85)	78 (63.4)	Reference	
> 1 cm	19 (15)	45 (36.6)	3.28 (1.81, 6.15)	
Site				
Esophagogastric junction	104 (81.9)	112 (91.1)	2.25 (1.07, 5.01)	*0.038*
Gastrojejunal anastomosis	19 (15)	11 (8.9)	0.56 (0.25, 1.21)	0.147
EGJ + distal staple line (SG)	1 (0.8)	0 (0)		
Distal staple line (SG)	3 (2.4)	0 (0)		
Stent				
Time to stent (days) M ± SD	17.8 ± 4.5	23 ± 6.5	1.17 (1.12, 1.24)	<*0.001*
Type				
Niti-S				
18 cm	10 (7.9)	14 (11.4)	1.50 (0.65, 3.62)	0.349
23 cm	57 (44.9)	43 (35)	0.66 (0.40, 1.10)	0.110
Hanaro				
18 cm	17 (13.4)	8 (6.5)	0.45 (0.18, 1.06)	0.075
21 cm	43 (33.9)	58 (47.2)	1.74 (1.05, 2.92)	*0.033*

Odds ratio representing likelihood of stent failure with one unit change in numeric predictor or presence of categorical predictor. Italicized values indicate statistical significance, *CI* confidence interval, *Inf* infinity, *EGJ* esophagogastric junction, *SG* sleeve gastrectomy, *M* ± *SD* mean ± standard deviation

Medical conditions significantly associated with failure included OSA (OR = 6.00), diabetes (OR = 2.96), hepatomegaly (OR = 3.04), hypertension (OR = 1.99), and hyperlipidemia (OR = 1.88).

Failure was also significantly associated with larger leaks (>1 cm) (OR = 3.28), esophagogastric junction leaks (OR = 2.25), while gastrojejunal anastomosis leaks appeared more frequent in success cases but did not reach statistical significance. Mean time from diagnosis to stent placement was significantly associated with failure (OR = 1.17 per day delay), as was the Hanaro 21 cm stent (OR = 1.74).

### Comparison Between Model Development and External Validation Cohorts

The validation sample displayed significantly lower BMI, diabetes prevalence, and stent failure, but a higher prevalence of OSA, PCOS, smaller leaks (<1 cm), distal SG leaks, and longer time to stent placement and duration of stent use (Supplementary Table 2). There were no differences in all the other variables under examination.

### Performance of Machine Learning Algorithms After Hyperparameter Optimization

Supplementary Table 3 summarizes the performance of the tested machine learning algorithms. Linear support vector machine (SVM) achieved 0.92 test AUC, followed by neural network (0.89), then logistic regression models (Lasso, Ridge, and Elastic Net) achieving 0.87 test AUC each. Although the linear SVM and neural network exhibited slightly higher discriminative ability, the Lasso logistic regression was selected for risk score derivation due to its competitive performance and greater clinical interpretability.

### Classification Performance of the Final Lasso Logistic Regression Model on External Validation Dataset

Performance of the final Lasso logistic regression model on the external validation dataset achieved 82% overall accuracy, high precision (0.91) for predicting stent success, and moderate precision (0.67) for stent failure (Supplementary Table 4). The metrics also indicated good sensitivity in identifying at-risk patients, balanced classification performance, and robust overall performance. Supplementary Table 5 depicts the performance metrics of the linear SVM and neural network models for comparison, supporting the selection of the Lasso model for risk score development due to its stable and interpretable performance across both outcome classes.

### Permutation-Based Feature Importance From the Final Lasso Model

**Fig. 2:**
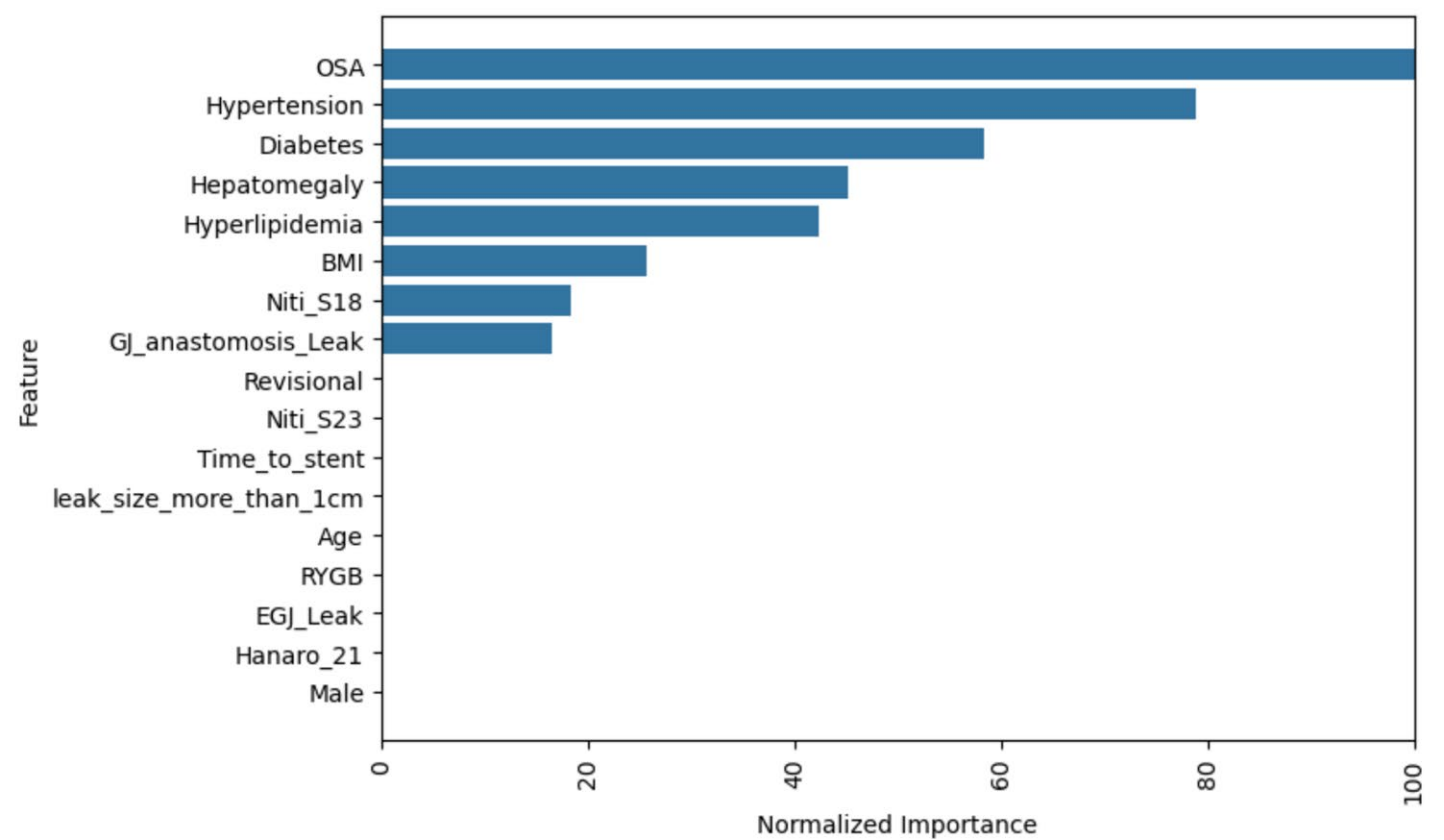
Permutation-based feature importance derived from the final Lasso logistic regression model. *Note*: Importance scores reflect the relative contribution of each predictor to the model’s ability to distinguish between stent success and failure, normalized to a scale of 0–100. Higher values indicate greater influence on model predictions. OSA, obstructive sleep apnea; BMI, body mass index; GJ, gastrojejunal; EGJ, esophagogastric junction; RYGB, Roux-en-Y gastric bypass

Figure 2 shows that the most influential predictors of stent failure comprised associated medical conditions including OSA, hypertension, and diabetes, each displaying the highest normalized importance scores, emerging as the strongest contributors to the model’s predictive performance. Hepatomegaly, hyperlipidemia, and BMI also ranked moderately high, indicating a potential role in risk stratification. In contrast, stent type (Niti-S18 and Niti-S23), anastomotic site of leak (GJ anastomosis leak), revisional surgery, and time to stent implantation demonstrated relatively lower importance. Features such as leak size >1 cm, age, type of surgery (RYGB), EGJ leak, use of Hanaro 21 stent, and sex (male) showed minimal contribution to the model’s predictions, suggesting less influence on the decision function within this series.

### Development of Point-Based Risk Scoring System

Table 3 presents the conversion of the Lasso logistic regression coefficients into a point-based scoring system for predicting stent failure. The strongest contributors to the total score were diabetes (74 points), OSA (72 points), hepatomegaly (65 points), hyperlipidemia (63 points), and hypertension (61 points)—reflecting their substantial positive association with stent failure. Moderate contributors included GJ anastomosis leak and EGJ leak (31 points each), Niti-S18 stent (14 points), and Hanaro 21 stent (10 points).

**Table 3:** Conversion of Lasso model coefficients into point-based scoring system for predicting stent failure

Predictor	Coefficient	Raw points^a^	Rounded points
Diabetes	2.42	73.84	74
Obstructive sleep apnea	2.36	72.10	72
Hepatomegaly	2.14	65.37	65
Hyperlipidemia	2.07	63.23	63
Hypertension	2.01	61.37	61
Gastrojejunal anastomosis leak	1.01	30.79	31
Esophagogastric junction leak	1.01	30.76	31
Niti-S18	0.45	13.83	14
BMI	0.42	12.79	Use BMI formula^b^
Male	−0.36	−11.00	−11
Hanaro 21	0.31	9.54	10
Leak size >1 cm	−0.17	−5.32	−5
Roux-en-Y gastric bypass	−0.16	−5.03	−5
Age	−0.05	−1.63	Use age formula^c^
Revisional surgery	0.03	1.00	1

Base coefficient = 0.03 (revisional surgery)

^a^Each predictor assigned raw score by dividing its coefficient by absolute value of base coefficient (raw points = coefficient ÷ base coefficient), then rounding to nearest whole number

^b^BMI points: [BMI (kg/m^2^) − 47.2]/3.6 × 12.79 (formula converts BMI [continuous variable] into risk score points using standardization and scaling)

^c^Age points: [Age (years) − 44.26]/8.23 × 1.63 (formula converts age [continuous variable] into risk score points using standardization and scaling)

Negative point values were assigned to male sex (−11 points), leak size >1 cm (−5 points), and RYGB (−5 points), indicating an inverse relationship with failure risk. BMI and age were retained as continuous predictors and scored using formulas derived from their respective coefficients.

Table 4 presents the clinical interpretation of total risk score values derived from the point-based Lasso model. Score ranges were grouped into meaningful risk categories based on estimated probabilities of stent failure. A total score ≤7 indicated very low risk (<1%), while scores ≥198 indicated an extremely high failure risk (>96%). In-between increasing scores signify increasing failure risk. This stratification allows clinicians to interpret scores in practical terms and prioritize interventions for high-risk patients.

**Table 4:** Risk categories and estimated probability of stent failure based on total risk

Score range	Estimated risk of failure	Risk level
≤7	<1%	Very low
8–47	1–5%	Low
48–77	5.1–15%	Moderate
78–117	15.1–50%	High
118–157	50.1–83%	Very high
158–197	83.1–96%	Severe
≥198	>96%	Extremely high

### External Validation of Risk Score Model

**Fig. 3:**
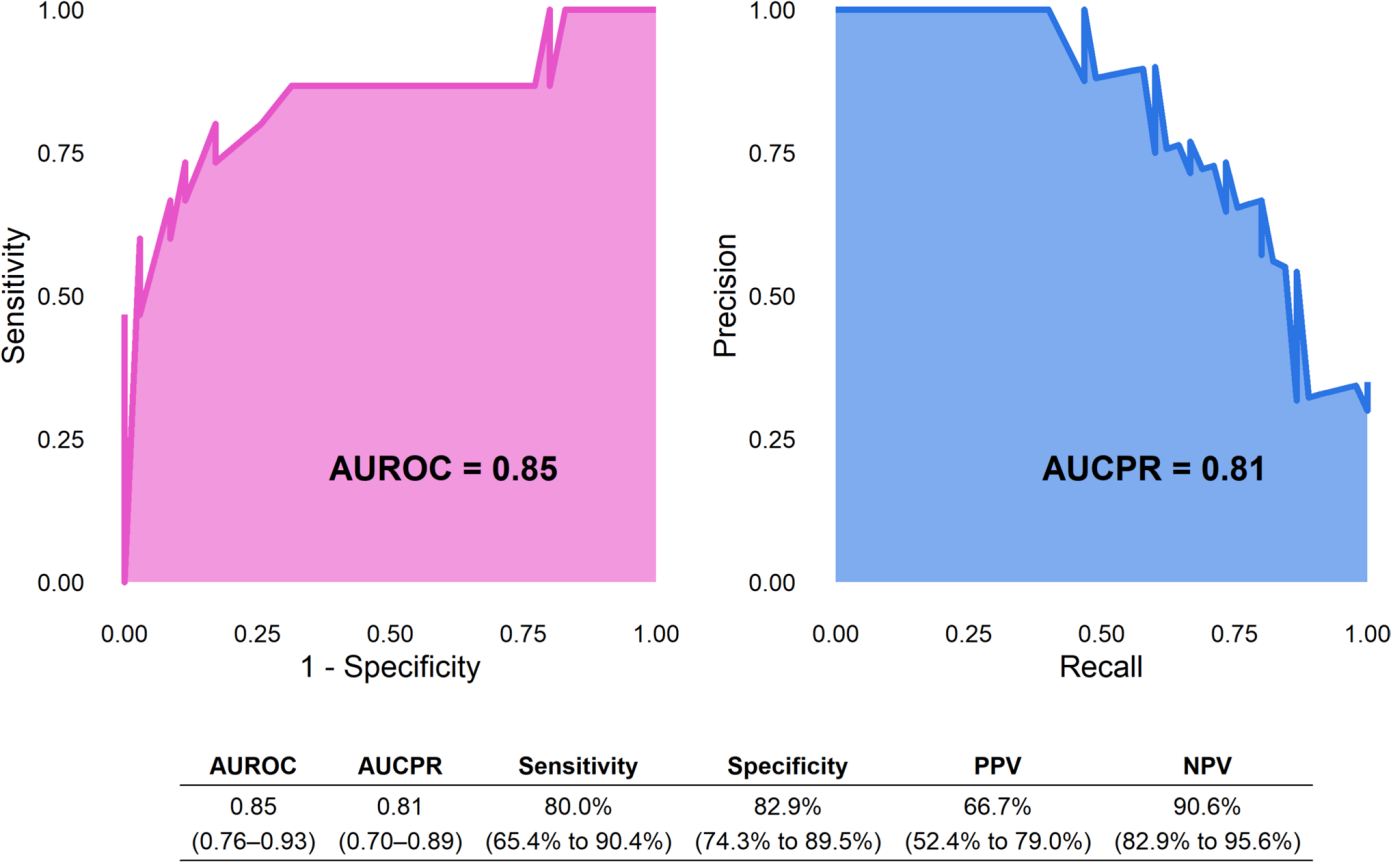
Performance of the risk score model in the external validation set. The left panel shows 0.85 area under the receiver operating characteristic curve (AUROC), indicating good discrimination. Precision-recall (PR) curve (right panel) shows 0.81 area under the curve (AUCPR), reflecting robust predictive power, particularly in the positive class. The table below the panels summarizes diagnostic metrics with 95% confidence intervals, including sensitivity (80.0%), specificity (82.9%), positive predictive value (66.7%), and negative predictive value (90.6%)

The predictive performance of the finalized risk score model was evaluated using the external validation dataset (Fig. 3). The model’s 0.85 AUROC indicated good overall discriminative ability, and its 0.81 AUCPR reflected strong performance in identifying true stent failures while accounting for class imbalance. At the optimal cutoff, the model yielded 80.0% sensitivity, 82.9% specificity, 66.7% PPV, and 90.6% NPV.

### Calibration of Risk Score Model in Validation Cohort

**Fig. 4:**
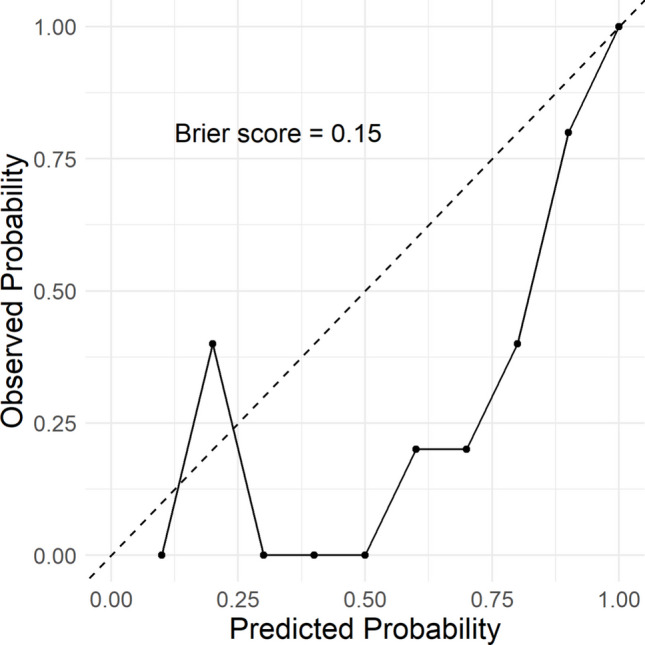
Calibration plot of the point-based risk score in the external validation cohort (*n* = 150). *Note*: Dashed line represents perfect calibration; solid line shows observed probability of stent failure across deciles of predicted risk. Brier score measures the mean squared difference between predicted probabilities and actual outcomes, ranging from 0 (perfect accuracy) to 1 (poor accuracy); a Brier score of 0.15 reflects acceptable calibration (reasonable agreement) between predicted and observed stent failure rates in the validation cohort

The calibration performance of the point-based risk score model using the external validation dataset (Fig. 4) showed that while deviations were evident at lower predicted probabilities, the model demonstrated improved calibration as predicted risk increased, particularly in the mid-to-high probability range. Brier score was 0.15, indicating acceptable overall calibration accuracy.

### Clinical Utility Assessment Using Decision Curve Analysis

**Fig. 5:**
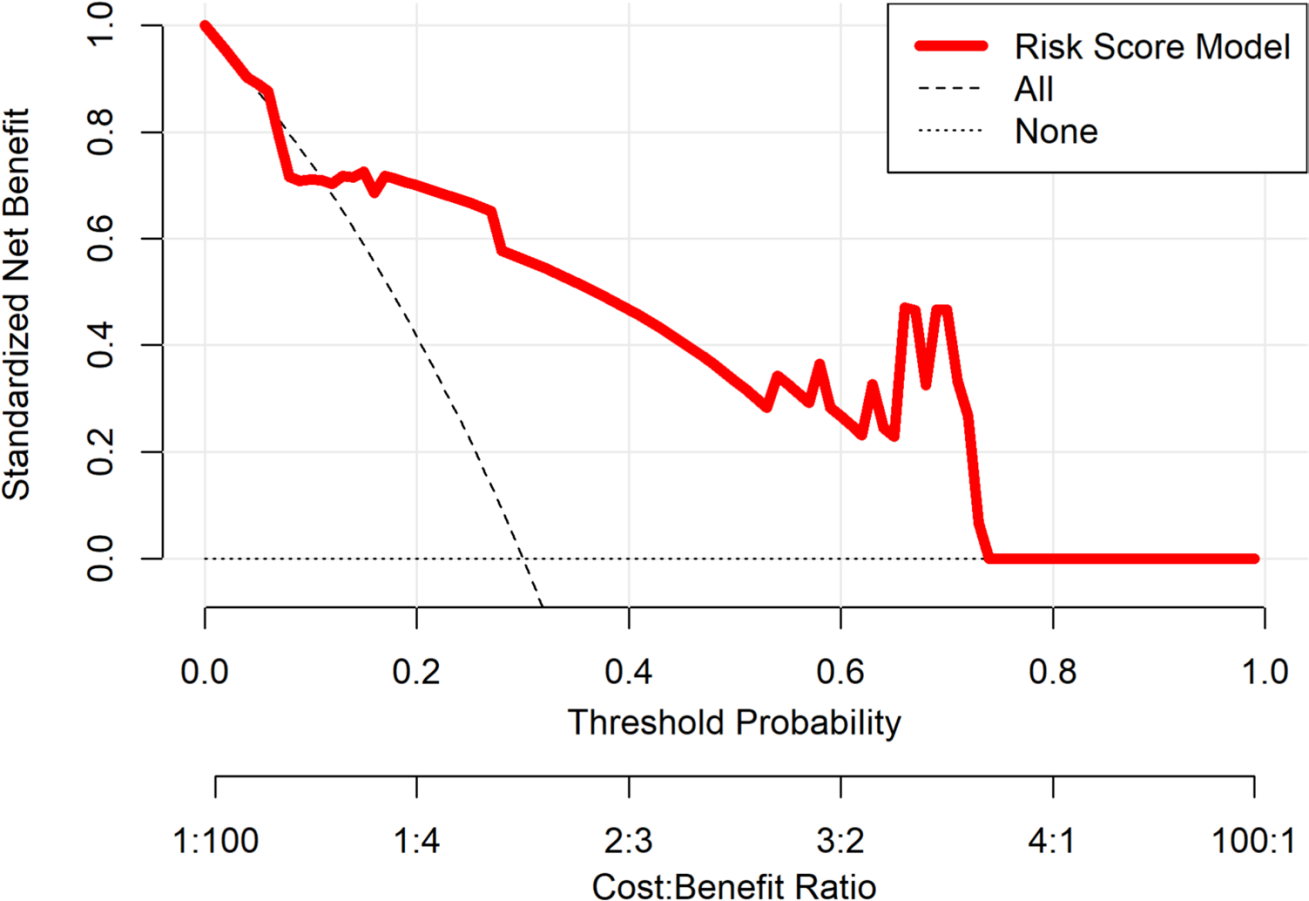
Decision curve analysis showing standardized net benefit of point-based risk score model in external validation cohort. *Note*: Solid red line represents net benefit of using machine learning model to guide clinical decisions across range of threshold probabilities, compared with two default strategies: “treating all patients” (dashed line) and “treating none” (horizontal dotted line at zero). The model offers greater net benefit across a range of clinically relevant threshold probabilities (0.10–0.80)

DCA evaluated the clinical utility of the point-based risk score model in the external validation dataset (Fig. 5). The model exhibited a higher net benefit than both extremes across a broad range of threshold probabilities, approximately between 0.10 and 0.80, suggesting that applying it in clinical practice could result in more effective decision-making than uniformly treating all patients or not treating any patients, particularly for intermediate-risk individuals. These findings underscore the model’s practical value in identifying patients at highest risk of stent failure who might benefit from closer monitoring or alternative management.

### Clinical Outcomes and Subsequent Interventions in Patients Undergoing Stent Placement for Post-MBS Leak

**Fig. 6:**
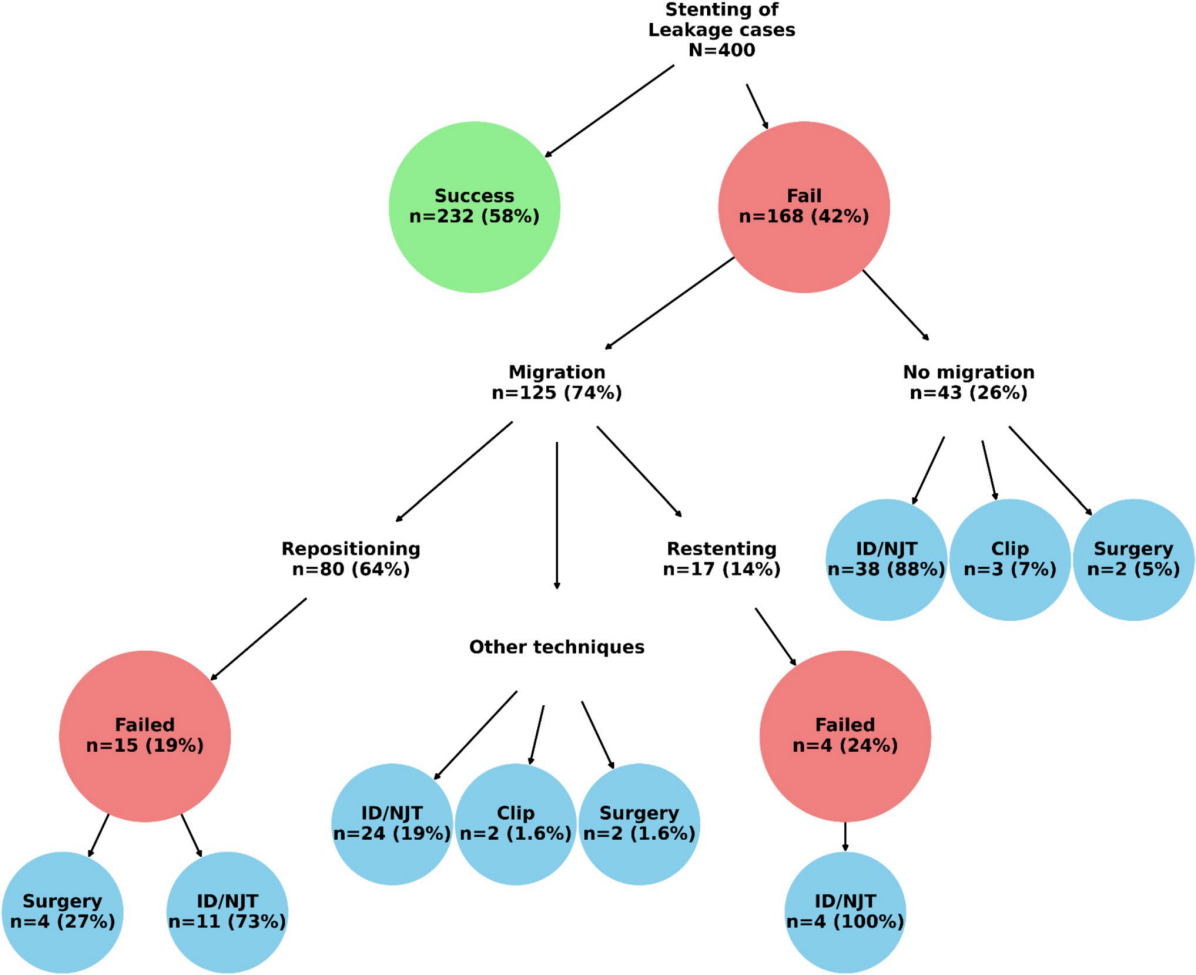
Clinical outcomes of stent placement for post-MBS leaks and subsequent management following failure (combined development and validation cohorts, *N* = 400). Note: The figure illustrates success and failure rates, stent migration, and distribution of secondary interventions including repositioning, re-stenting, internal drainage/nasojejunal tube (ID/NJT) placement, clips, and surgery. Percentages calculated within each pathway branch

Figure 6 provides a visual summary of the clinical pathways and outcomes among the combined development and validation cohorts who underwent stenting for post-MBS leaks (*N* = 400). Stent implantation was successful in 232 cases (58%) but failed in 168 cases (42%). Among the failures, 125 patients (74%) experienced stent migration, while 43 patients (26%) experienced stent failure without migration. For those with migration, the most common management approach was repositioning (*n* = 80; 64%), which resulted in 15 residual failures (19%) managed by either surgery (27%) or internal drainage/nasojejunal tube (ID/NJT) placement (73%). Additional strategies in migrated cases included re-stenting (*n* = 17; 14%) and other techniques such as ID/NJT, clips, and surgery, each applied in a small proportion of patients. Four cases failed re-stenting and were managed exclusively with ID/NJT. Among the 43 patients without migration, the most frequent post-failure intervention was ID/NJT (88%), followed by clips (7%) and surgery (5%). The flowchart highlights both the heterogeneity of post-failure management and the dominance of ID/NJT as a fallback intervention across different scenarios.

## Discussion

This study developed and externally validated a novel ML-based risk score (Alexandria-Bari-Stent) to predict stent failure in post–bariatric leaks. The model demonstrated excellent discrimination (AUROC 0.85) and calibration in an independent cohort identifying high-risk patients. It was particularly effective at ruling out potential stent failure: patients classified as low failure risk had ≈91% NPV, indicating that the model reliably identified individuals unlikely to experience failure. This high NPV is clinically valuable, supporting more confident decision-making in deferring unnecessary interventions or follow-up testing for low-risk patients. This externally validated tool (Alexandria-Bari-Stent) is the first in the MBS leak setting.

Post-MBS leakage ensues without recognizable technical problems during the procedure and is the second cause of postoperative mortality [26, 27]. Anticipating stent failure risk is key to individualized treatment. However, there are no clinically feasible post-MBS stent failure risk prediction models to accurately forecast failure, no consensus on the endoscopic management of such leaks, and no data to support a precise algorithm [28, 29].

In response, the current study developed the Alexandria-Bari-Stent, a clinical post-MBS ML-based risk model to predict stent failure for leaks, employing 250 patients from one MBS center, and externally validated on 150 patients from another center. We also evaluated the model’s discrimination, calibrated it against observed outcomes, and assessed its clinical utility using DCA.

Our main findings were that the significant predictors of post-MBS stent failure comprised eight high contributors (OSA, hypertension, diabetes, hepatomegaly, hyperlipidemia, BMI, Niti-S18 stent, GJ anastomosis leak) and nine features with varying contributions (revisional surgery, Niti-S23 stent, time to stent implantation, leak size >1 cm, age, RYGB surgery, EGJ leak, Hanaro 21 stent, male sex). External validation demonstrated the diagnostic performance in predicting stent failure (0.85 AUROC and 0.81 AUCPR), indicating good discriminative ability and strong performance in identifying true stent failure cases while accounting for class imbalance. The model’s 80.0% sensitivity and 66.7% PPV indicated reasonable ability in identifying patients at risk of stent failure. Its 82.9% specificity and 90.6% NPV meant it was particularly effective at identifying patients unlikely to fail. Clinically, the model is more reliable for ruling out stent failure than for confirming it, especially useful in reassuring low-risk leakage patients. The calibration illustrated reasonable agreement between the predicted and observed failure rates, with a Brier score of 0.15 indicating acceptable calibration accuracy, and improved calibration as predicted risk increased, particularly in the mid-to-high probability range.

The absence of predictive models of post-MBS stent failure for leak renders head-to-head comparisons of our model vis-a-vis others unfeasible. However, examination of other published ML-based MBS prediction models highlights the favorable findings of the current study, demonstrating its rigorous approach to the development, testing, and calibration of the Alexandria-Bari-Stent’s diagnostic performance.

Existing applications of ML in MBS have reported moderate predictive power for various outcomes, e.g., AUROCs of 0.77 for liver fibrosis in severe obesity [30], 0.65–0.7 and 0.64–0.68 for postoperative complications [31, 32], 61–64% for complications in conversion surgery [33], and 0.67–0.78 for readmissions [34–36]. Our model’s performance (AUROC 0.85, 95% CI: 0.76–0.93) was notably high and, more importantly, one of the very few with true external validation.

The current study externally validated the risk score model on stent patients from another center. Despite that external validation is the “ideal,” many ML studies in MBS did not undertake or report it, conducting internal validation instead [30–32, 34–40]. Internal validation is insufficient to substantiate that a model that successfully predicts the outcome of interest is valuable or applicable to new individuals, particularly since predictive models tend to predict observations in the derived dataset more accurately than in new data [41, 42]. A further attestation to the robustness of the current study’s model is our higher accuracy compared to other published ML models, despite the fact that we externally validated the model when others undertook internal validation [30, 31, 37].

Few if any bariatric ML studies reported calibration [30–32, 34–40, 43]. We demonstrated acceptable calibration (Brier ~0.15), meaning that the predicted risk corresponds well to actual risk, which is important for a clinical decision-making tool and critical in clinical prediction models [44].

The mean age, BMI, and female majority of the patients employed in our model development concur with a meta‐analysis of stent management for post-MBS leaks [1], as well as with studies of post-SG leaks undergoing endoscopic stenting [45]. Our observed 1.2% mortality was close to the 1.4% mortality reported elsewhere [27].

Few studies assessed the characteristics associated with stent failure. We found associated medical problems (diabetes, hypertension, hyperlipidemia, OSA) to be strongly associated with stent failure. Similarly, others observed higher complication rates after bariatric stenting among patients with diabetes, reaching up to a fourfold increased risk [21, 46]. However, a smaller series did not find diabetes predictive [43]. Endoscopic stents simultaneously manage leaks and strictures when present [21, 47–49], and the self-expandable metal stents we used are designed with the capability of leak sealing and stricture dilation functions.

We noted that BMI influenced stent failure, concurring with that stent migration was significantly more frequent with higher BMI [46]. Although some studies reported that age was not associated with failure [21, 50], males were more represented in our failure cohort. While sex has not been widely reported as a failure factor [21, 50], this finding might reflect underlying risk profiles and warrant further investigation.

The time to stent implantation was a contributor to failure in the current series, reinforcing the importance of early intervention [4, 17, 46, 51]. Within our study, each additional day of delay increased the failure odds by ~17%. Timely stent placement is a key modifiable factor for success.

Previous studies have not consistently linked the type of surgery to stent outcomes. Our analysis found that RYGB was associated with a modestly lower risk of stent failure in both univariable and multivariable models, although the association was not statistically significant [50]. Interestingly, our model suggested that stent use for leaks occurring after revisional procedures might have a higher failure propensity, probably due to more complex anatomy or ensuing fibrosis. However, with limited literature, this needs corroboration.

We found that some stents (Niti-S18 and, to a lesser extent, Niti-S23 and Hanaro 21) were associated with failure. Some authors have advocated for larger “mega” stents to improve success [10], but our findings, similar to others, suggest that bigger is not necessarily better [29]. Stent choice in our study was not randomized, and optimal stent choice depends on the patient’s anatomy, leak characteristics, and availability, underscoring the need for further research on stent design and selection.

Pertaining to stent failure rates, among our combined development and validation cohorts (*N* = 400), placement was successful in 232 cases (58% primary closure) and failed in 168 cases (42%); 74% of the failures were because of stent migration (125 patients). A challenge when comparing post-MBS stent failure rates across studies is how success/failure is defined and reported and whether success/failure is calculated based on the first stenting or after multiple subsequent endoscopic maneuvers that ultimately result in success (after initial failure) [52]. We selected a stringent definition of failure (failure to resolve leak with the initial stent) to avoid conflating outcomes of additional interventions. Systematic reviews of stent management of post-MBS leaks reported failure and success rates, but with unclarity whether these were calculated based on first (initial) or further (subsequent) stenting [1]. Standardized definitions of stent success or failure as well as their preferred reporting are required for valid comparisons across studies, MBS techniques, time, and countries [52].

An observation that the current study noted is the importance of the patient’s general health status to the outcomes of post-MBS stent, above the traditional leak-related factors and delayed diagnosis/treatment. Some predictors of failure we noted pertained to the medical problems including OSA, hypertension, diabetes, hyperlipidemia, and hepatomegaly. With no published prediction models, we are unable to compare our findings; however, low nutritional reserves impair wound healing, and these conditions are all components of the metabolic syndrome and obesity-related co-morbidity cluster. Mechanistic hypotheses for such associations are plausible: OSA causes chronic intermittent hypoxia and poor sleep, impairing wound healing; diabetes/associated hyperglycemia leads to poor circulation and immune dysfunction, slowing tissue repair; hypertension is accompanied by vascular changes that reduce tissue perfusion and is often part of a broader metabolic syndrome; hyperlipidemia contributes to a pro-inflammatory state; elevated triglycerides can undermine postoperative healing by promoting systemic inflammation; and hepatomegaly and advanced obesity/metabolic syndrome are potentially associated with chronic inflammation and coagulopathy that could hinder leak resolution [6, 53–63].

Our model’s top predictors are complementary to, not in conflict with, established leak management principles. The variability in patients’ comorbid profiles could have contributed to the model capitalizing on it for prediction. Conversely, nearly all our patients received relatively prompt treatment (median ~20 days from diagnosis), providing the model with less variability to capitalize upon when predicting. In summary, metabolic health mattered in addition to standard care, and host factors are important in leak outcomes in addition to established technical and local factors. In this sense, the model places a spotlight on the patient’s baseline condition that can tilt the balance between success and failure of the same treatment, and that more aggressive adjunctive therapy of associated medical conditions can support the healing of leaks. A more holistic view is required, with a focus not only on the leak but also on patient optimization, as systemic factors play a larger role than previously recognized. This metabolic stabilization and the medically optimized patient as part of comprehensive care that the current model uncovered represent a shift from primary attention on the leak itself, as the perfusion of surrounding tissues, general patient condition/nutrition, and infection affect the healing of post-MBS leaks [19].

The current study has limitations. The study is retrospective and non-randomized; patients were from two centers, stent choice was not randomized, and patient characteristics and postoperative management may not reflect practices at other centers using different stents or management protocols. Hence, generalizing the model’s performance and findings to wider populations needs to be cautious. Hanaro 18 and 21 stents were excluded from the regression model as they did not have a minimum of 10 failures each. The inclusion of other variables that could influence stent outcomes would have been useful, e.g., nutritional parameters (e.g., albumin/prealbumin), sepsis at presentation, bougie size at index operation (tighter sleeve [small bougie] could predispose to leaks), presence of distal obstruction, and endoscopist’s decision-making or stent allocation strategy. We did not systematically document concurrent endoscopic interventions such as balloon dilation for strictures; however, the stents we used can manage leaks and strictures simultaneously, which may have influenced our success rates. The absence of a standardized, validated leak classification system during our study period limited our ability to apply established criteria, highlighting a need for practical and validated classification systems that correlate with clinical outcomes. In addition, nutritional management protocols varied between centers, with decisions individualized based on leak characteristics, patient stability, and institutional preferences reflecting real-world clinical practice. The specific breakdown of TPN versus enteral feeding approaches was not systematically documented in our retrospective analysis. Finally, our model is intended for use in patients selected for endoscopic stent management of leak; it does not address which leaks should be stented or initially managed surgically. As all patients in our study received a stent, the score presumes an initial decision to stent has been made. It can help identify when a stent is likely to fail—but it does not replace clinical judgment in the initial choice of therapy. A point-based score, while convenient, is a simplification. There may be some loss of granularity, and it might not perfectly calibrate in every setting; thus, further prospective validation is needed. Future studies should validate the risk score in larger, multi-center cohorts across different regions to ensure broad applicability and incorporate the Alexandria-Bari-Stent tool into prospective decision-making protocols, possibly testing it in a clinical trial where high-risk patients are triaged to alternative strategies to demonstrate impact on patient outcomes. Prospective iterations should incorporate nutritional markers and operative technique details to further enhance the predictive modeling and document all concurrent interventions to better characterize the full scope of endoscopic management.

Despite these limitations, the study has many strengths. We analyzed the demographic, surgical, clinical, leak, and stent-related factors associated with stent failure; compared the performance of ML algorithms, selecting the most appropriate; evaluated the performance of the Lasso model on the external validation dataset; assessed the model’s permutation-based feature importance; converted the Lasso coefficients into a point-based scoring system for predicting stent failure; externally validated the risk score model on another 150 patients; calibrated the performance of the point-based risk score model in the external validation dataset; and appraised its clinical utility using DCA. We also generated a flowchart of the clinical outcomes of stent placement and subsequent management after failure across 400 post-MBS leaks. To our knowledge, this is the first study to undertake this comprehensive task. To expand on the clinical utility of the Alexandria-Bari-Stent tool, we are developing a multitask platform that would help surgeons in clinical decision-making to reduce stent failure risk.

## Conclusion

We developed an externally validated risk score that accurately predicts stent failure in post–bariatric leaks. The tool is meant to be used at the point of treatment planning (stenting), particularly for patients with higher predicted risk. The Alexandria-Bari-Stent tool has the potential to improve clinical decision-making by identifying patients who may not benefit from endoscopic stenting, thereby personalizing and optimizing leak management. Future studies should aim to include larger, more diverse cohorts to enhance the model’s generalizability. Ongoing evaluations of the Alexandria-Bari-Stent tool’s impact in clinical practice would provide further insights into its practical benefits and limitations.

## Supplementary Information

Below is the link to the electronic supplementary material.

ESM 1 (DOCX 32.9 KB)

## Data Availability

Data is available upon reasonable request and with the agreement of the institutions where the research was implemented.
