# Supplementary material for: Development and External Validation of a Machine Learning–Based Risk Score for Stent Outcomes in Post–Bariatric Leak Management: The “Alexandria-Bari-Stent” Tool
Source: Obes Surg. 2025 Nov 29;35(12):5120–35. doi: 10.1007/s11695-025-08321-6 (PMC12722429; doi:10.1007/s11695-025-08321-6)
Supplement: Supplementary file 1 — (DOCX 32.9 KB) [file 11695_2025_8321_MOESM1_ESM.docx]

**Supplementary Box 1.** Variable selection, data preprocessing, and machine learning

| **Variable Selection for Model Creation:** Univariable logistic regression evaluated the association between each predictor and stent failure. To minimize overfitting, each predictor had to have been observed in at least 10 failure events. Variables with insufficient failure events e.g., previous sleeve gastrectomy, osteoarthritis, and PCOS, were excluded. After such exclusions, 17 candidate variables were retained for model development [RYGB, revisional surgery, EGJ leak, GJ anastomosis leak, Niti S23, Niti S18, Hanaro 21, leak size >1 cm, hypertension, diabetes, hyperlipidemia, OSA, hepatomegaly, male sex, age, BMI, and time to stent] |
| --- |
| **Data Preprocessing for Machine Learning:** Numeric variables standardized using z-score normalization to ensure comparable scale and to enhance model performance. Categorical variables with two levels converted into binary indicators; for those with >2 levels, one-hot encoding created binary dummy variables for each level, while omitting one level to serve as reference category and avoid multicollinearity |
| **Machine Learning Process**  **Modeling**: Machine learning modeling conducted using scikit-learn library (version 1.4.2) in Python (version 3.12.3). Primary outcome was stent failure, modeled as binary variable. Eleven algorithms were tested. Model performance evaluated using AUROC, and best set of hyperparameters for each algorithm was selected. Hyperparameters explored are summarized in Supplementary Table 1. Each model’s final performance was evaluated by calculating test AUC using an external validation dataset. PPV and NPV provided for final model.  **Algorithms**: Among the tested algorithms, linear support vector machine and neural network models achieved slightly higher test AUROCs. However, Lasso logistic regression demonstrated comparably strong performance across all key evaluation metrics. This was selected as final model for interpretation and clinical application, given the small difference in its discriminative ability but added advantages of model transparency, coefficient sparsity, and ease of translating results into a point-based risk score  Permutation-based feature importance evaluated contribution of each variable to model's predictive accuracy. Variables with non-zero coefficients in Lasso model were used to develop point-based clinical risk scoring system, with points assigned in proportion to each variable’s standardized coefficient, scaled relative to the smallest non-zero coefficient. For continuous variable ‘Time to stent’ (standardized during modeling), risk score points computed based on number of standard deviations above or below mean and translated back to raw scale for clinical usability. Resulting scoring system applied to external validation dataset.  AUCPR complemented AUROC, appraised model’s ability to identify failure cases (i.e., positive class). As stent failure occurred in only 30% of external validation dataset, AUCPR provides more information on performance in this moderately imbalanced context, where accurately identifying true positives is clinically critical  **Calibration**: Model calibration assessed via calibration plot comparing predicted vs. observed risk and quantified with Brier score (0=perfect prediction, 0.25=no skill for binary outcomes).  **Decision curve analysis**: evaluated model’s clinical net benefit across threshold probabilities (treating all or no patients) |

*PCOS* polycystic ovary syndrome; *OSA* obstructive sleep apnea; *BMI* body mass index; *EGJ* esophagogastric junction; *GJ* gastrojejunal; *AUROC* area under the receiver operating characteristic curve; *PPV* Positive predictive values; *NPV* negative predictive values; *AUCPR* Area under precision-recall curve

**Supplementary Table 1.** Hyperparameters included in the tuning process with grid search

| **Classifier** | **Hyperparameters** |
| --- | --- |
| K-nearest neighbors (KNN) | Number of neighbors: 3, 5, and 7 |
| Logistic Regression |  |
| Lasso | C: 0.01, 0.1, 1, and 10 |
| Ridge | C: 0.01, 0.1, 1, and 10 |
| ElasticNet | C: 0.01, 0.1, 1, and 10; l1 ratio: 0.01, 0.1, 1, and 10 |
| Support vector machines |  |
| Linear | C: 0.1, 1, and 10 |
| Kernelized | C: 0.1, 1, and 10, gamma: 0.1, 1, and 10 |
| Naïve Bayes | None |
| Decision Tree | Maximum tree depth: 3, 5, and 7 |
| Ensemble |  |
| Random Forest | Number of trees: 50, 100, and 200 |
| Gradient Boosted | Number of trees: 50, 100, and 201 |
| Neural Network | Number of hidden layers sizes: 10, 50, and 100, activation: relu, tanh |

**Supplementary Table 2.** Comparison between development and validation samples

| **Characteristic** | **Development sample** | **Validation sample** | **p** |
| --- | --- | --- | --- |
| Demography | n=250 | n=150 |  |
| Sex |  |  | 0.700 |
| Male | 79(31.6) | 51(34) |  |
| Female | 171(68.4) | 99(66) |  |
| Age, mean±SD | 44.3±8.2 | 43.3±8.3 | 0.275 |
| Surgery |  |  |  |
| Index operation |  |  | 0.067 |
| Sleeve gastrectomy | 201(80.4) | 132(88) |  |
| Roux-en-Y gastric bypass | 49(19.6) | 18(12) |  |
| Type of surgery |  |  | 0.074 |
| Primary | 220(88) | 141(94) |  |
| Revisional | 30(12) | 9(6) |  |
| Previous surgery |  |  |  |
| Sleeve gastrectomy | 9(3.6) | 0(0) | *0.045* |
| Vertical banded gastroplasty | 15(6) | 6(4) | 0.524 |
| Lap band | 4(1.6) | 3(2) | 1.000 |
| Gastric plication | 1(0.4) | 0(0) | 1.000 |
| Clinical |  |  |  |
| Body mass index, mean±SD | 47.2±3.6 | 45.4±4.3 | *< 0.001* |
| Associated medical conditions |  |  |  |
| Hypertension | 63(25.2) | 36(24) | 0.881 |
| Diabetes | 84(33.6) | 21(14) | *< 0.001* |
| Hyperlipidemia | 73(29.2) | 51(34) | 0.372 |
| Obstructive sleep apnea | 85(34) | 75(50) | *0.002* |
| Polycystic ovary syndrome | 2(0.8) | 30(20) | *< 0.001* |
| Osteoarthritis | 6(2.4) | 24(16) | *< 0.001* |
| Hepatomegaly | 80(32) | 36(24) | 0.111 |
| Presenting symptoms |  |  |  |
| Fever | 250(100) | 150(100) | **—** |
| Raised C-reactive protein | 250(100) | 150(100) | **—** |
| Pain | 128(51.2) | 81(54) | 0.660 |
| Pleural effusion | 122(48.8) | 69(46) | 0.660 |
| Leak |  |  |  |
| Site |  |  |  |
| Distal staple line (SG) | 3(1.2) | 27(18) | *< 0.001* |
| Esophagogastric junction | 216(86.4) | 117(78) | *0.041* |
| Esophagogastric junction + Distal staple line (SG) | 1(0.4) | 0(0) | 1.000 |
| Gastro-jejunal anastomosis | 30(12) | 6(4) | *0.012* |
| Size |  |  | *< 0.001* |
| < 1 cm | 69(27.6) | 99(66) |  |
| > 1 cm | 181(72.4) | 51(34) |  |
| Stent |  |  |  |
| Time to stent placement (days), mean±SD | 20.3±6.2 | 25.5±6.5 | *< 0.001* |
| Duration of stent, (days), mean±SD | 21.8±6.9 | 26.2±5.1 | *< 0.001* |
| Type |  |  |  |
| Niti S |  |  |  |
| 18 cm | 24(9.6) | 9(6) | 0.280 |
| 23 cm | 100(40) | 57(38) | 0.771 |
| Hanaro |  |  |  |
| 21 cm | 101(40.4) | 75(50) | 0.077 |
| 18 cm | 25(10) | 9(6) | 0.229 |
| Stent failure | 123(49.2) | 45(30) | *<0.001* |
| Intensive care unit admission | 64(25.6) | 39(26) | 1.000 |
| Surgery | 5(2) | 6(4) | 0.385 |
| Hospitalization overall (days), mean±SD | 3.4±2.4 | 3.1±3.1 | 0.463 |
| Number of readmissions, median (range) | 1(1-4) | 1 (1-4) | 0.575 |
| Mortality | 3(1.2) | 3(2) | 0.832 |

Cell values represent frequency and percentages n(%) unless otherwise stated; *M±SD* mean±standard deviation; *SG* sleeve gastrectomy; italicized cells indicate statistical significance; **—** not applicable

**Supplementary Table 3.** Results of the grid search process with hyperparameter tuning

| **Classifier** | **Best hyperparameters** | **Training AUC** | **Test AUC** |
| --- | --- | --- | --- |
| K-nearest neighbors (KNN) | Number of neighbors: 7 | 0.80 | 0.71 |
| Logistic Regression |  |  |  |
| *Lasso* | *C: 10* | *0.81* | *0.87* |
| Ridge | C: 10 | 0.81 | 0.87 |
| ElasticNet | C: 10; l1 ratio: 0.1 | 0.81 | 0.87 |
| Support vector machines |  |  |  |
| **Linear** | **C: 0.1** | **0.81** | **0.92** |
| Kernelized | C: 1, gamma: 0.1 | 0.80 | 0.83 |
| Naïve Bayes | None | 0.78 | 0.81 |
| Decision Tree | Maximum tree depth: 3 | 0.77 | 0.50 |
| Ensemble |  |  |  |
| Random Forest | Number of trees: 100 | 0.79 | 0.69 |
| Gradient Boosted | Number of trees: 100 | 0.78 | 0.56 |
| Neural Network | Number of hidden layers sizes: 10, activation: relu | 0.80 | 0.89 |

Shaded row indicates model achieved the highest performance; *AUC* area under curve; *KNN* K-nearest neighbors; *SVM* support vector machines; Although bolded row (Linear) represents the best performing model, the italicized row (Lasso) was the final model used, as its performance was very close to the Linear, and in addition it can be used for derivation of risk scores

**Supplementary Table 4.** Performance metrics of Lasso model on external validation dataset (n=150)

| **Label** | **Precision** | **Recall** | **F1-Score** | **Support** |
| --- | --- | --- | --- | --- |
| 0 (Success) | 0.91 | 0.83 | 0.87 | 105 |
| 1 (Failure) | 0.67 | 0.80 | 0.73 | 45 |
| Accuracy |  |  | 0.82 | 150 |
| Macro Avg | 0.79 | 0.81 | 0.80 | 150 |
| Weighted Avg | 0.83 | 0.82 | 0.82 | 150 |

Note: Recall for stent failure=0.80 (good sensitivity in identifying at risk patients); F1-score=0.87 for success, 0.73 for failure (indicating balanced classification performance); macro-average F1-score=0.80, and weighted average=0.82 (confirming robust overall performance).

**Supplementary Table 5**. Performance metrics of Support vector machines model and Neural network on external validation dataset (n=150)

| **Label** | **Precision** | **Recall** | **F1-Score** | **Support** |
| --- | --- | --- | --- | --- |
| Support vector machines |  |  |  |  |
| 0 (Success) | 0.86 | 0.89 | 0.87 | 105 |
| 1 (Failure) | 0.71 | 0.67 | 0.69 | 45 |
| Accuracy |  |  | 0.82 | 150 |
| Macro Avg | 0.79 | 0.78 | 0.78 | 150 |
| Weighted Avg | 0.82 | 0.82 | 0.82 | 150 |
| Neural network |  |  |  |  |
| 0 (Success) | 0.95 | 0.51 | 0.67 | 105 |
| 1 (Failure) | 0.45 | 0.93 | 0.61 | 45 |
| Accuracy |  |  | 0.64 | 150 |
| Macro Avg | 0.70 | 0.72 | 0.64 | 150 |
| Weighted Avg | 0.80 | 0.64 | 0.65 | 150 |

Note: Compared to Lasso logistic regression model, linear Support vector machines model achieved comparable accuracy (82%), slightly higher precision for failure (0.71), and lower recall (0.67); neural network model demonstrated high sensitivity for failure (0.93 recall) but reduced precision and overall accuracy (64%), reflecting a performance balance trade-off
